# Micronutrient Supplementation in Frailty: A Systematic Review and Meta-Analysis of Randomized Controlled Trials

**DOI:** 10.3390/healthcare13222828

**Published:** 2025-11-07

**Authors:** Themistoklis Paraskevas, Konstantinos Kotrokois, Tonia Vassilakou, Panagiotis Halvatsiotis, Theodora Psaltopoulou, Pavlos Sarafis, Theodoros N. Sergentanis

**Affiliations:** 1 Department of Nephrology, University Hospital of Patras, 26504 Patras, Greece; themispara@hotmail.com; 2 Department of Public Health Policy, School of Public Health, University of West Attica, 12243 Athens, Greece; kkotrokois@uniwa.gr (K.K.); tvasilakou@uniwa.gr (T.V.); tsergentanis@uniwa.gr (T.N.S.); 3 2nd Propaedeutic Department of Internal Medicine, School of Medicine, “Attikon” University Hospital, National and Kapodistrian University of Athens, 11527 Athens, Greece; pahalv@gmail.com; 4 Department of Clinical Therapeutics, Medical School, “Alexandra” Hospital, National and Kapodistrian University of Athens, 15771 Athens, Greece; tpsaltop@med.uoa.gr; 5 Department of Nursing, University of Thessaly, 41222 Volos, Greece

**Keywords:** frail elderly, vitamin D, micronutrients, functional status, falls

## Abstract

**Highlights:**

**What are the main findings?**

- The quality of published evidence for vitamin D and multicomponent supplementation is low or very low for most outcomes, and clinicians should be aware of this fact before prescribing them.
- Frailty is inconsistently measured in published clinical trials.

**What are the implication of the main findings?**

- Future research should focus on patient-oriented outcomes, such as changes in frailty levels, functional status, and cognitive function.
- Providers should be aware of the low certainty of evidence when prescribing micronutrient supplements.

**Abstract:**

**Background/Objectives**: Low levels of vitamins and minerals are linked to increased frailty, but the effectiveness of micronutrient supplementation remains debated. **Methods**: A systematic search of PubMed and Embase (end of search 10 June 2025) identified 21 randomized controlled trials from 33 articles assessing supplementation in frail individuals. **Results**: Regarding vitamin D supplementation, seven studies (2600 participants) reported all-cause mortality (pooled RR: 1.04, 95% CI: 0.83 to 1.31, I^2^ = 35%) with moderate certainty of evidence, whereas only one study reported on the change in frailty levels. For multicomponent supplementation, four studies (180 participants) were identified on all-cause mortality, and two studies on change in frailty levels (pooled MD = −0.28, 95% CI: −0.71 to 0.16, I^2^ = 0%) with very low certainty of evidence for both outcomes. Only one study investigated nicotinamide supplementation. **Conclusions**: Further research is needed to justify the prescription of micronutrient supplementation in this population. Future research in frailty should focus on longitudinal change in frailty levels, cognitive function, and functional measures.

## 1. Introduction

Frailty syndrome is defined as a clinically recognizable state in the older population that is associated with increased vulnerability resulting from decreased physiological reserves and multi-organ dysfunction, which leads to the inability to cope with everyday and acute stressors [1]. Frailty syndrome has become an emerging threat in modern societies, and the ever-growing, ageing population highlights the need for change in public health policies. The occurrence of frailty differs between countries and settings, but it is estimated that one in six community-dwelling older people may be frail [2]. Additionally, pre-frailty, a state associated with increased risk of morbidity and frailty development, is prevalent in the older population. The prevalence of pre-frailty is reported to be as high as 40% in older adults in Germany [3].

Vitamins and minerals are essential nutrients that are required in small amounts for the proper function of biological organisms. Older adults commonly use oral supplements, either self-prescribed or under medical supervision. These include single micronutrients, such as vitamin D, or multicomponent formulations containing vitamins and minerals, including B, C, and E vitamins, folate, selenium, and magnesium. The cost of vitamin D supplements in the UK has increased from 13 to 111 million pounds over the past 18 years, and their effectiveness is often not certain [4]. According to the National Institute for Health and Care Excellence (NICE) in the United Kingdom, oral multivitamin and mineral supplements should help individuals who are eating poorly to meet their vitamin and mineral requirements [5].

Vitamin D, in particular, has received attention for its potential role in frailty due to its broad physiological effects on muscle, bone, and immune function. Low serum vitamin D levels have been linked to frailty, possibly through impaired muscle protein synthesis and heightened inflammation [6]. Although evidence exists regarding the association between micronutrient deficiency and the incidence of frailty, the effects of supplementation interventions in pre-frail and frail older adults have not been systematically investigated. The evidence specifically addressing this population and synthesizing results across different micronutrients remains limited and inconclusive.

The aim of this systematic review is to determine the effect of micronutrient supplementation on measures of physical frailty and related outcomes in frail and pre-frail older adults. This review specifically addressed the following PICO question: among frail and pre-frail older adults, does micronutrient supplementation, either single or multicomponent, compared with placebo or no supplementation, improve measures of physical frailty and related outcomes such as falls, fractures, functional status, or mortality?

## 2. Materials and Methods

### 2.1. Eligibility Criteria

#### 2.1.1. Types of Studies

This systematic review has been registered in PROSPERO (CRD42022372266). There, the data analysis plan and the approach to assessing quality and certainty of evidence can be found. This systematic review was conducted in line with the PRISMA guidelines and a PRISMA checklist has been filled (Supplementary Files). The following supporting information can be downloaded at: https://www.mdpi.com/article/10.3390/healthcare13222828/s1: PRISMA 2020 checklist. While conducting this review, there were no deviations from the published protocol, other than updating the final search date. We included randomized controlled trials (RCTs), cluster-RCTs, and quasi-RCTs. Non-randomized studies, editorials, letters, commentaries, and cross-sectional studies were excluded from this systematic review.

#### 2.1.2. Types of Participants

We included frail and pre-frail individuals irrespective of their housing conditions (resident homes, community-dwelling, etc.). As there is no single gold standard in the diagnosis of frailty, we considered all studies that described their population as frail eligible for this review. Studies that investigated the role of supplements in acute conditions (e.g., SARS-CoV-2 infection) were excluded.

#### 2.1.3. Types of Interventions (And Co-Interventions)

In this systematic review, we expected to find two types of interventions: either supplementation with a single vitamin or micronutrient or with a formulation including various vitamins and micronutrients. These two types of interventions (single-vitamin and multicomponent) were analyzed separately. Additionally, we expected that some studies would investigate the effect of supplementation in combination with or combined with exercise. We included studies in which exercise was provided in both arms and was considered a background treatment, and excluded studies in which only one of the arms received an exercise intervention.

We included studies that used a comparator group receiving a placebo, no intervention, or a different dose of the supplement.

### 2.2. Search Strategy

We conducted a comprehensive search on PubMed/Medline and Embase using the following terms: (vitamin* OR mineral* OR lycopene OR ascorbic OR tocopherol OR retinol OR folate OR carotenoid* OR betacarotene OR selenium OR pyridoxine OR iron) AND (“frail elderly” [MeSH Terms] OR “frail*” OR “frailty”). The end-of-search date was 10 June 2025. Although only studies published in English were eligible for inclusion, no studies were excluded on the basis of language.

### 2.3. Study Selection and Data Extraction

Screening and data extraction were performed by two authors independently. Conflicts were resolved by a third independent author. Deduplication was performed using the Covidence online software tool. For each included study, the following data were extracted: first author, year of publication, population, interventions, comparisons, definition of frailty, relevant outcomes (all-cause mortality, frailty levels, falls, fractures, muscle strength, gait speed, body mass measures, cognitive function, inflammatory markers, physical performance), and effect sizes. Additionally, for each study, we abstracted information regarding randomization, blinding, intervention, and stratification procedures.

### 2.4. Risk of Bias Evaluation and GRADE Assessment

Risk of bias assessment was performed independently by two authors using the Cochrane Risk of Bias 1 tool [7]. We used the GRADE approach to assess the certainty of evidence of primary and secondary outcomes, starting from high certainty for RCTs and downgrading based on the trials’ limitations [8].

### 2.5. Statistical Synthesis

For each primary or secondary outcome that was reported by at least two studies, quantitative synthesis was performed using random-effect meta-analysis. Random effects were used considering the expected clinical heterogeneity of the populations in frailty studies, the definition of frailty, and different types of intervention. For categorical and continuous outcomes, risk ratios (RRs) and mean differences (MDs) with 95% confidence intervals were calculated, respectively. Statistical analysis was performed with RevManWeb software. Additionally, we tabulated qualitative and quantitative data by constructing tables summarizing study characteristics and a summary of findings table.

## 3. Results

### 3.1. Results of the Search

**Figure 1:**
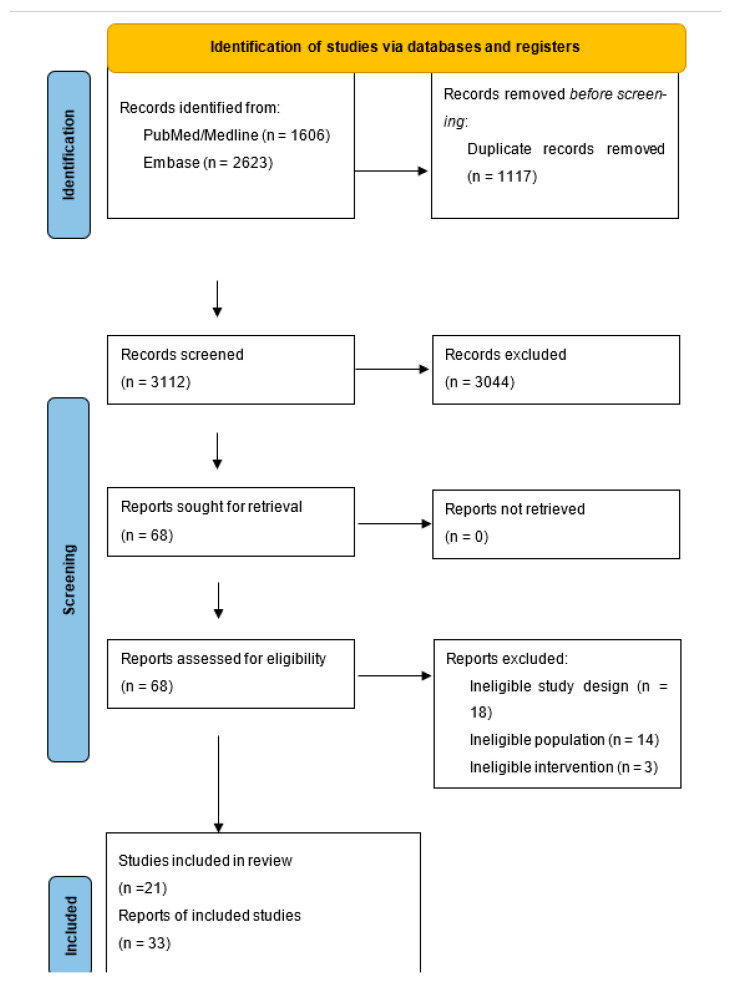
PRISMA flow diagram illustrating the study selection process for the systematic review.

Our search in PubMed/Medline and Embase resulted in 1606 and 2623 records, respectively. After removal of duplicates, a total of 3112 items were screened, resulting in 68 potentially eligible reports. Eighteen reports were excluded due to ineligible study design (non-randomized studies, study protocols), 14 due to ineligible population, and three due to ineligible intervention. Finally, we included 21 studies described in 33 reports (Figure 1). A summary of findings of the included studies can be found in Table 1.

### 3.2. Included Studies

We identified 11 studies (16 reports) investigating the effect of vitamin D supplementation in frail older individuals [9,10,11,12,13,14,15,16,17,18,19,20]. Seven of these studies used an established frailty scale to assess participants, five used Fried’s Frailty Scale [9,12,15,19,20], one used the Winograd Criteria [13], and one used the Frail Elderly Functional Assessment [14]. The majority of included studies administered daily doses of vitamin D analogs (cholecalciferol, ergocalciferol, calcidiol, alphcalcidiol). Most of these studies used between 400 IU and 1000 IU per day, ranging from 200 IU to 4000 IU in the dose-finding STURDY trial [12]. Two older studies used very high doses of vitamin D supplementation (Gloth 1995: 400 IU per day to 100,000 IU every 3 months and Latham 2003 [13]: single 300,000 IU dose). The characteristics of included studies regarding vitamin D supplementation are shown in Supplemental Table S1.

Additionally, we found nine studies (15 reports) on the effect of multicomponent supplementation in frail older individuals [21,22,23,24,25,26,27,28,29]. Only two of them used an established frailty scale, namely the Fried’s Frailty Scale [23,24]. Different formulations and enriched products were identified. A detailed description of the studies on multicomponent supplementation can be found in Supplemental Table S2.

We found a single published randomized study reporting on the effects of nicotinamide supplementation on frail elderly (using Fried’s Frailty Scale) at a daily dose of 250 mg [30].

### 3.3. Risk of Bias in Included Studies

**Figure 2:**
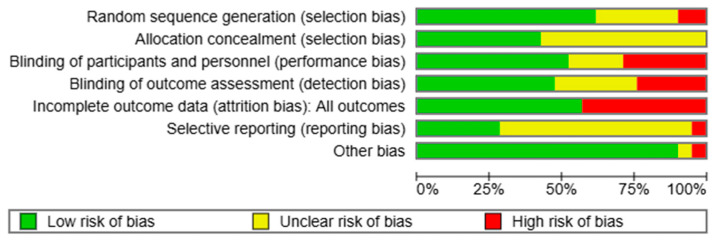
Summary of risk of bias across included studies, showing the overall distribution of bias judgments for each domain.

The overall quality of studies was moderate. 14 out of 21 studies had at least one domain with high risk of bias, and only two studies had low risk of bias in all domains [9,20]. A graphical presentation of the risk of bias is shown in Figure 2 and Figure S1. Detailed assessments and justifications for each individual study are provided in Supplemental Tables S3 and S4.

*Randomization and Allocation*: Out of 11 studies on vitamin D supplementation, eight had low, two had unclear, and one had a high risk of randomization bias. We considered the randomization method in the study by Meyer et al. to be insufficient, as they divided patients into groups based on date of birth [18]. Allocation methods were not described adequately in six out of nine studies [11,12,13,14,16,17,18].

Regarding the studies on multicomponent supplementation, five of nine did not provide sufficient details on randomization, and six of nine lacked adequate information on allocation concealment [21,22,24,25,26,27,28,29].

The nicotinamide study was considered to be at a high risk of selection bias due to serious imbalances in baseline data between groups [30].

*Blinding*: Overall, blinding methods (participants, personnel, and outcome assessors) were judged to be adequate in studies on vitamin D supplementation. Only one study had a high risk of bias [10] as participants and researchers were not blinded to the intervention. One study had an unclear risk of bias (blinding methods for participants, personnel, and outcome assessors were not described in text) [14], and one had an unclear risk only regarding blinding of outcome assessment [16].

On the other hand, studies investigating multicomponent supplementation mostly had a high risk of detection and/or performance bias (five out of nine studies). The remaining studies had an unclear risk of bias in at least one of the two domains [21,22,23,25,26].

Blinding was considered adequate in the nicotinamide study [30].

*Incomplete outcome data*: Three studies on vitamin D supplementation [10,16,18] and five studies on multicomponent supplementation [21,22,25,26,29] had a high risk of attrition bias, as less than 85% of randomized participants were analyzed in these studies. The nicotinamide study was considered to be at a low risk of bias in this domain [30].

*Selective reporting*: The study protocol was not available for six vitamin D [10,11,13,14,16,18] and eight multicomponent supplementation studies [21,22,24,25,26,27,28,29], all of which were judged to have an unclear risk of bias in this domain. We considered the study by Rizka et al. to be at high risk of bias, as the incidence of respiratory tract infection, the main outcome in the registered protocol, was not reported [15]. The nicotinamide study was considered at a low risk of bias in this domain [30].

*Other potential sources of bias*: One included study had an unclear risk of other bias, as the supplemented dose and frequency were changed during the course of the study, and one study was considered at a high risk of bias as it was terminated early due to low recruitment rates [11,19]. Reported conflicts of interest and funding for each can be found in the Supplementary Tables S3 and S4.

### 3.4. Vitamin D Supplementation

#### Outcomes

1. Vitamin D Supplementation: All-cause mortality

**Figure 3:**
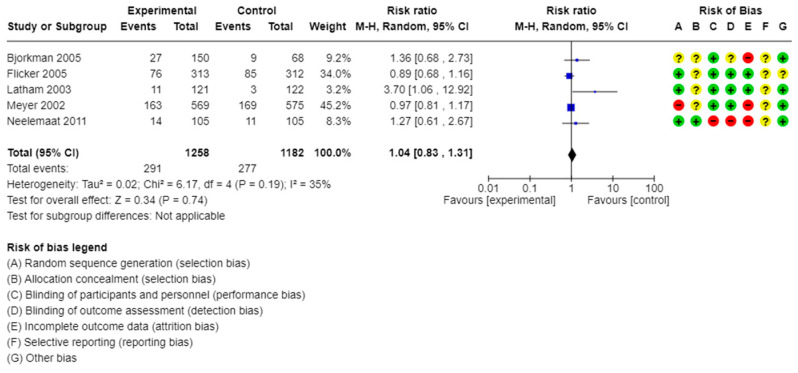
Meta-analysis of studies evaluating the effect of vitamin D supplementation on all-cause mortality in frail individuals [10,11,13,16,18]. yellow?: unclear; green+: low risk of bias; red−: high risk of bias.

Seven studies with a total of 2600 participants reported the effect of vitamin D supplementation on mortality. The studies by Rizka et al. and Vaes et al. followed participants for 3 and 6 months, respectively, and did not report any deaths during the trials’ period [9,15]. Compared to placebo or no intervention, vitamin D supplementation probably resulted in little to no difference in mortality (pooled RR: 1.04, 95% CI: 0.83 to 1.31, I^2^ = 35%, Figure 3). The certainty of evidence was moderate; we downgraded one point for high risk of bias, mainly in the trial by Meyer [18].

2. Vitamin D Supplementation: Frailty levels, as measured by validated frailty scales, such as the Frailty Index and Fried’s Frailty Phenotype

Two studies investigated the effect of vitamin D supplementation on frailty levels. In the trial by Appel et al., there was no difference between pooled higher doses (1000 IU/d, 2000 IU/d, 4000 IU/d) and the control dose (200 IU/d) in risk of frailty incidence, frailty worsening, or improving [12]. Interestingly, during the dose-finding phase of the trial, the 2000 IU/d group had a higher risk of worsening frailty levels (hazard ratio (HR)  =  1.89, 95% CI: 1.13–3.16, *p*  =  0.015), while the 4000 IU/d dose had a lower risk for developing frailty (HR  =  0.22, 95% CI: 0.05–0.97, *p*  =  0.045). Those spurious differences might be attributed to baseline vitamin D status. On the other hand, Gloth et al. did not report the comparison between the two arms [14].

3. Vitamin D Supplementation: Falls

**Figure 4:**
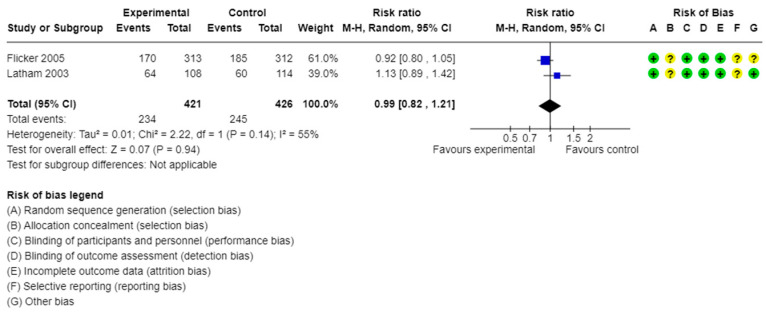
Meta-analysis of studies assessing the effect of vitamin D supplementation on the number of frail participants experiencing at least one fall [11,13]. yellow?: unclear; green+: low risk of bias.

We identified three studies investigating the number of participants with at least one fall. Each study considered a different dose of vitamin D supplementation, ergocalciferol 1000 IU/d, a single oral dose of 300,000 IU, and high doses of cholecalciferol 1000–4000 IU/d [11,12,13]. The latter used a low dose of 200 IU/d in the control group [12]. None of those studies found a significant effect of supplementation on the number of people with at least one fall. Vitamin D supplementation did not reduce falls in frail individuals (pooled RR = 0.99, 95% CI: 0.82 to 1.21, I^2^ = 55%, Figure 4, Grade: Moderate, downgrade 1 point for inconsistency).

The study by Bischoff-Ferrari et al. was not included in the meta-analysis (as RR could not be calculated by the available data), but reported no significant differences between the 24,000 IU vitamin D3/month group versus placebo (OR = 2.243, 95% CI: 0.891, 5.650, *p* = 0.086) [19].

4. Vitamin D Supplementation: Fractures, including hip fractures and vertebral fractures, among others

Fractures were reported by three studies. Compared to the other two studies, Meyer et al. investigated a relatively lower dose of intervention of cholecalciferol (440 IU/d), but there were no significant differences between the intervention and the control group in any of the studies [11,12,18]. Compared to placebo, vitamin D supplementation may result in little or no difference in fractures in frail older individuals (pooled RR: 0.77, 95% CI: 0.59 to 1.01, I^2^ = 0%, Supplemental Figure S2, Grade: low, downgrade 1 point for wide CI, 1 point for risk of bias).

5. Vitamin D Supplementation: Muscle strength as measured by handgrip strength

Dwimartutie et al., Meyer et al., Neelemaat et al., and Vaes et al. investigated the change in handgrip strength after 12 weeks, 12, 3, and 6 months of supplementation, respectively [8,10,18,20]. All studies had null findings. This outcome was available in a small number of participants of the trial by Meyer et al. (*n* = 51) in a secondary report [27]. In the quantitative synthesis, the two arms in the Vaes et al. study (10 mcg/d of 25(OH)D3 and 20 mcg/d of cholecalciferol) were combined. Vitamin D supplementation probably leads to little or no change in handgrip strength (pooled MD: −0.03, 95% CI: −0.87 to 0.81, I^2^: 10%, Figure S3, Grade: moderate, downgrade 1 point for imprecision due to low number of participants).

6. Vitamin D Supplementation: Gait speed

Regarding change in gait speed, Appel et al. did not find significant differences between the higher pooled doses group vs. the low dose group after 3 and 6 months of supplementation, but the higher doses provided a small but significant protective effect after 12 months of supplementation (MD = 0.06 m/s, 95% CI: 0.02–0.10) [12]. There was a significant reduction from baseline after 6 and 12 months of supplementation in both arms. In the study by Dwimantutie et al., walking speed decreased by 0.05 m/s in both the experimental and control groups compared to baseline, and no between-group difference was reported [20].

Vaes et al. followed patients up to 6 months and did not find any significant differences between the three arms (25OHD3, cholecalciferol, and placebo) regarding gait speed [9]. Similar to Appel et al., there was a within-group decrease from baseline during the study period. Bischoff-Ferrari et al. found a non-significant increase in the change in gait speed in participants receiving vitamin D versus placebo [19].

7. Vitamin D Supplementation: Body mass measures, including lean mass, fat-free mass, total mass, and body mass index.

Two studies reported changes in body mass index [9,10]. Neelemaat et al. did not find any significant differences in fat-free mass after 3 months of supplementation, but in subgroup analysis, there was a significant increase in participants with weights > 63.9 kg at baseline (MD 3.4, 95% CI: 0.2–6.6) [10]. Similarly, in the trial by Vaes et al., supplementation with vitamin D for 6 months did not result in a change in total lean mass compared to placebo [9]. Bischoff-Ferrari found that vitamin D had no effect on appendicular lean mass [19].

8. Vitamin D Supplementation: Cognitive function as measured by validated scales such as the Mini-Mental State Exam

No studies reported this outcome.

9. Vitamin D Supplementation: Inflammatory markers, including but not limited to cytokines and CRP

Bjorkman et al. did not find any significant differences after the administration of different doses of cholecalciferol versus placebo in either CRP (*p* = 0.523) or fibrinogen (*p* = 0.184). The observed extreme changes in CRP (e.g., −96.75 to 395.62 in the 1200 IU/d arm) might be attributed to other factors, such as acute inflammatory procedures [16].

In the trial by Rizka et al., supplementation with 0.5 mcg alphcalcidiol daily for 90 days resulted in significant changes in IL-10 levels, IL-6/IL-10 ratio, CD4/CD8 ratio, and CD8+ CD28- percentage. Those changes suggested a shift towards a more anti-inflammatory state [15].

In the study by Dwimantutie et al., daily cholecalciferol supplementation did not significantly affect IL-6 or IGF-1 expression compared to the placebo group or baseline values [20].

10. Vitamin D Supplementation: Functionality

Latham et al. reported several measures of functionality, including the Barthel Index, the Adelaide Activities Profile, and the Medical Outcomes Study 36-item Short Form (SF-36), but did not find any significant differences 3 months after a single high-dose vitamin D dose [13]. Likewise, in the Neelemaat et al. study, 3-month supplementation with 400 IU of vitamin D did not lead to significant between-group changes in functional limitation score, physical performance score, or physical limitation score [10].

Bischoff-Ferrari reported two measures of functional performance, the Short Physical Performance Battery test and the five-times sit-to-stand test. There was no difference in either measure in the 12 months of follow-up between participants receiving vitamin D or placebo (*p* = 0.5 and 0.302, respectively) [19].

### 3.5. Multicomponent Supplementation

1. Multicomponent Supplementation: All-cause mortality

Mortality was not reported in the majority of the studies, and only two deaths (one in each group) were reported in the study by Imaoka [21]. Thus, we are very uncertain about the effect of multicomponent supplementation on mortality in frail individuals.

2. Multicomponent Supplementation: Frailty levels, as measured by validated frailty scales, such as the Frailty Index and Fried’s Frailty Phenotype

**Figure 5:**
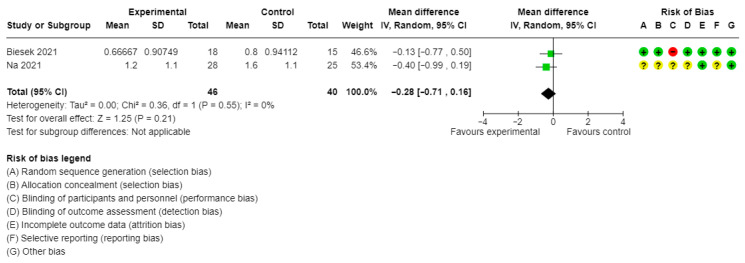
Meta-analysis of studies summarizing the pooled effect of multicomponent supplementation on frailty levels in frail individuals [23,24]. yellow?: unclear; green+: low risk of bias; red−: high risk of bias.

Two studies with 86 participants evaluated the effect of multicomponent supplementation on frailty measured with Fried’s Frailty Phenotype (pooled MD = −0.28, 95% CI: −0.71 to 0.16, I^2^ = 0%, Figure 5) [23,24]. The evidence was very uncertain about the effect of multicomponent supplementation on frailty levels (Grade: Very low, downgrade 2 for imprecision and 1 for risk of bias).

3. Multicomponent Supplementation: Falls

Only one study investigated the effect of multicomponent supplementation on falls [21]. While the researchers did not find significant differences between either the supplementation or the exercise group compared to the placebo, the group that received both interventions had a significantly lower hazard compared to placebo (adjusted for sex and age, HR = 0.276, 95% CI: 0.083–0.924, *p* = 0.037).

4. Multicomponent Supplementation: Fractures, including hip fractures and vertebral fractures, among others

No studies reported this outcome.

5. Multicomponent Supplementation: Muscle strength as measured by handgrip strength

Four studies with a total of 153 participants were included in the meta-analysis (pooled MD = 0.76, 95% CI: −1.35, 2.87, I^2^ = 31%, Supplemental Figure S4 [21,23,24,27]. Across all four studies, no statistically significant differences were detected for this outcome. De Jong et al. did not find significant differences between the supplementation and placebo group, but were not included in the meta-analysis as they reported the median, 10th, and 90th percentile values [26]. The effect of multicomponent supplementation on frailty levels is very uncertain (Grade: Very low, downgrade 2 for imprecision and 1 for risk of bias)

6. Multicomponent Supplementation: Gait speed

Abe et al. analyzed changes after 3 months of supplementation and found a significant difference between the three arms. The group receiving multicomponent supplementation that included medium-chain acids showed a slight increase compared to baseline [27]. The other two groups (supplementation that included long-chain fatty acids and control) showed a small decline from baseline. It must be noted that there was a high attrition rate in this outcome (only 24 participants analyzed in total) and that the medium-chain acids group had numerically higher walking speed at baseline. Due to those differences, we did not pool the data from the two intervention groups.

In the study by Bonnefoy et al., supplementation did not have a significant effect on 6 m walk time at 3 and 9 months [22]. Similarly, in the trial by de Jong et al., changes from baseline were similar for the intervention and the control group (0.0 ± 0.1 and 0.1 ± 0.1 m/sec, respectively) [26].

7. Multicomponent Supplementation: Body mass measures, including lean mass, fat-free mass, total mass, and body mass index

Five studies reported the effect of multicomponent supplementation on body mass-related indices [22,23,24,26,27]. We performed two separate meta-analyses for BMI (pooled MD: 0.69, 95% CI: −0.78 to 2.16, I^2^ = 21%, Supplemental Figure S5) and body weight (pooled MD: 1.23, 95% CI: −0.91 to 3.37, I^2^ = 30%, Supplemental Figure S6). There is considerable uncertainty about the effect of multicomponent supplementation on both indexes (Grade Very Low, downgrade 2 for imprecision and 1 for risk of bias). The findings of Bonnefoy et al. were not included in the analysis, as they only reported % variation from baseline [22]. However, they found a significant increase in BMI compared to the control group after 3 and 9 months of supplementation using nutritional supplements consisting of proteins, carbohydrates, lipids, minerals, and vitamins twice daily.

8. Multicomponent Supplementation: Cognitive function as measured by validated scales such as the Mini-Mental State Examination

Two studies with a total of 89 participants reported on the effect of multicomponent supplementation on MMSE score (pooled MD: 1.34, 95% CI: −1.45 to 4.14, I^2^ = 0%, Supplemental Figure S7, Grade: Very low, downgrade 2 for imprecision due to low amount of events and participants and 1 for risk of bias) [24,27]. The evidence was very uncertain about the effect of multicomponent supplementation on the MMSE score.

Wouters-Wesseling et al. investigated several memory tests and found significant differences in the word learning test and category fluency (professions) but not in the delayed word learning test, recognition memory test for words, or category fluency (animals) [29]. This study was at high risk of bias due to attrition, and these inconsistent findings require further validation.

Regarding dementia, there was a significant difference in the Nishimura Geriatric Scale between the intervention groups (nutritional supplementation and different forms of fatty acids) and the control group (MD: 9.10, 95% CI: 5.12 to 13.08) [27]. Imaoka et al. did not find a significant effect on Hasegawa’s Dementia Scale after 3 months of supplementation [21].

Gosney et al. evaluated the effect of multicomponent supplementation on mood scores (Hospital Anxiety and Depression Score, HADS, and Montgomery–Asberg Depression Rating Scale, MADRS) [25]. They reported a trend towards a positive effect on HADS score in the intervention group and, conversely, a trend towards a positive effect on HADS anxiety score in the control group. This study was prone to significant attrition bias and enrolled a small number of participants (59 participants were analyzed).

9. Multicomponent Supplementation: Inflammatory markers, including but not limited to cytokines and CRP

Biesek et al. did not find a significant change in IL-6 levels either between or within groups after 3 months of supplementation [23]. It must be noted that only 9 and 11 participants were analyzed in the control and protein supplementation group, respectively. Similar findings were reported by de Jong et al. for CRP (MD: 0.3, CI: 95%: −1.5 to 2.2) and ferritin (MD: −7, CI: 95%: −18 to 5), but according to the authors, CRP was calculated only in 11 participants. The exact number of participants who had a ferritin measurement was not reported [26].

10. Multicomponent Supplementation: Functionality

We identified three studies that reported several different measures regarding the effect of multicomponent supplementation on functionality. More specifically, Imaoka et al. reported on functional independence measures after 6 months, de Jong et al. on activities of daily living score, mobility score, and self-care score after 17 weeks, and Na et al. on activities of daily living after 3 months [21,24,26]. None of these between-group comparisons revealed a significant between-group difference.

### 3.6. Nicotinamide (Vitamin B3) Supplementation

We identified one published study that included 15 patients and investigated the effect of nicotinamide in pre-frail and frail diabetic individuals with a 24-week follow-up [30]. The investigators found no difference in any of their prespecified endpoints, including grip strength, walking speed, skeletal muscle mass index, and chair standing time. A trend of improvement in frailty levels is reported, but it should be interpreted with caution, as there was a serious imbalance in the baseline frailty scores between the two groups.

## 4. Discussion

We included a total of 21 studies (33 reports) that compared vitamin D, nicotinamide, or multicomponent supplementation to no treatment or placebo in frail older individuals. For outcomes with sufficient data to allow quantitative synthesis, we did not find significant differences between supplementation and control groups. The meta-analysis of two studies (1769 participants) reporting on fractures showed a trend towards lower fracture incidence in participants receiving vitamin D supplementation. Limited evidence from the literature suggests that vitamin D supplementation might lead to a more prominent anti-inflammatory state. It is important to note that in a considerable number of analyses, the confidence intervals were broad enough to include both possible benefit and harm, further contributing to the uncertainty of the synthesized findings. This effect was most apparent for fracture outcomes among patients receiving vitamin D supplementation, where the confidence interval slightly crossed the null value. However, the trend toward benefit remains consistent with clinical and biological expectations.

Our systematic review aimed to address a gap in the existing literature by focusing on an increasingly relevant population group, older adults defined by frailty status. Previous research has largely concentrated on the general older population or on specific outcomes, often without distinguishing levels of frailty. Consistent with our results, a recent systematic review by Prokopidis et al. did not find a positive effect in sarcopenic older adults after vitamin D monotherapy [31], in line with the results of controversial individual studies [32,33]. On the other hand, in pre-clinical models, evidence has shown that vitamin supplementation could be used to attenuate frailty [34]. Vitamin D supplementation in rats led to significantly lower frailty index compared to age-matched controls. Another study in mice reported increased physical frailty in vitamin D-deficient rats [35].

Vitamin and mineral deficiencies are common among frail older individuals. Despite the aforementioned lack of consistent evidence regarding supplementation, observational studies in humans have suggested a strong association between vitamin D deficiency and measures of physical frailty [36]. Low intake of vitamins B6, C, E, and folate has also been associated with a higher risk for frailty in a Spanish population study [37], and a greater severity of frailty was found in older hospitalized patients with vitamin C deficiency [38]. In addition, the inverse association between serum 25(OH)D concentration and frailty severity has made vitamin D analogs an attractive choice for further testing [39,40].

Similar to vitamins, other micronutrient deficiencies have been associated with frailty through modulation of inflammation, oxidative stress, and muscle and bone metabolism [41]. According to a systematic review of observational studies, higher dietary and plasma levels of carotenoids were associated with lower odds of frailty [42]. Regarding minerals, low selenium levels have been associated with mortality in frail older people, possibly due to pleiotropic effects, including protection from oxidative stress and inflammation [43]. Magnesium has also been implicated in frailty development, based on findings from a study in community-dwelling older Japanese women [44]. Recent observational evidence further supports the potential role of micronutrient status in frailty. In a large cross-sectional study including 4009 participants, higher dietary intakes of trace elements, chromium, manganese, copper, zinc, selenium, and iron, were associated with a decreased likelihood of frailty, as assessed by the FRAIL scale [45].

Malnutrition is common in the ever-growing older population and has been associated with increasing frailty and sarcopenia [46]. Ample research has been conducted to investigate the complex mechanisms underlying geriatric syndromes and malnutrition [47,48,49]. Although significant associations between micronutrient deficiency and geriatric frailty have been extensively reported, the effectiveness of supplementation remains uncertain [50,51,52]. The results of our review provide clinicians with evidence to support balanced decision-making regarding micronutrient supplementation in frail populations. Supplementation decisions should remain individualized, considering the high prevalence of vitamin D deficiency and its potential non-skeletal benefits, while recognizing the limited evidence for improvement in frailty-related outcomes. Clinicians should also educate patients about the limited and uncertain benefits of micronutrient supplementation, ensuring that expectations align with the current evidence base.

Considering the multifaceted nature of frailty, multicomponent interventions might be more appropriate than supplement monotherapy [53]. In this context, physical activity has been shown to improve physical performance in frail individuals, and combining these interventions might produce better results [54,55,56]. Of particular interest are two randomized trials that investigated the effect of micronutrient supplementation in robust adults on frailty trajectories [57,58]. The DO-HEALTH trial employed a factorial design, and while it found no effect in the vitamin D group, the researchers reported lower odds of becoming pre-frail in the group that received a combination of vitamin D, omega-3 supplementation, and home exercise. Respectively, an ancillary study of the VITAL 2 × 2 factorial trial, which included over 25,000 mostly robust adults, found that neither vitamin D nor omega-3 supplementation had a significant effect on mean frailty score or incidence of frailty. Conversely, a meta-analysis of 18 randomized controlled trials reported moderate to large beneficial effects of instability resistance training on both physical and cognitive function in older adults [59].

The grading of evidence was moderate only for all-cause mortality and falls in the analysis pertaining to vitamin D supplementation, while it was low or very low for all other outcomes. According to our findings, high-quality evidence on this subject is currently lacking. Potential misclassification of participants as frail due to not utilizing frailty scales might limit the generalizability of the findings in the included studies. We could not perform a priori subgroup analyses or construct funnel plots to detect differential effects and publication bias, respectively, because of the limited number of the included studies. The overall quality of evidence among the included studies was moderate to low, as 14 out of 21 studies had a high risk of bias in at least one domain.

Commenting on the strengths and limitations of our study, we performed a rigorous and systematic search and screening process designed to identify all relevant studies addressing micronutrient supplementation in frail and pre-frail populations. Because our search was restricted to two major databases and did not cover gray literature, we compensated for this by performing comprehensive snowball searches. Full-text manuscripts for all eligible studies were obtained and independently assessed. A key strength of our review is that we evaluated both patient-relevant clinical outcomes, such as frailty status, falls, fractures, and mortality, and laboratory or intermediate outcomes, providing a comprehensive understanding of the potential effects of micronutrient interventions. Furthermore, we applied the GRADE framework to rate the certainty of evidence for each outcome, allowing for transparent interpretation of the strength and reliability of our conclusions. However, the inclusion of studies that did not utilize an established frailty scale to diagnose frailty syndrome might limit the applicability of the results and introduce bias. Variability in how frailty was defined, ranging from formal diagnostic scales to proxy measures and clinical judgment, could have contributed to heterogeneity across studies and influenced the pooled estimates. Due to the low quality of available evidence, the results of this systematic review cannot support or refute the use of micronutrient supplementation in frail older people in clinical practice. Prior to prescribing such formulations, medical practitioners should inform their patients about the lack of strong evidence on the respective clinical outcomes. Future trials should include longer follow-up periods and consistently assess frailty levels as key outcomes to better determine the effectiveness of these interventions.

## 5. Conclusions

Vitamin D supplementation probably leads to little or no change in all-cause mortality and incidence of falls, while it may lead to little or no difference in fractures in frail individuals. Regarding multicomponent supplementation, the certainty of evidence was very low across both primary and secondary outcomes. Future research on frailty should incorporate additional frailty-related outcomes, such as longitudinal changes in frailty levels, cognitive function, and functional measures.

## Figures and Tables

**Table 1:** Summary of findings table presenting the effect of vitamin D and multicomponent supplementation on key outcomes in frail individuals, assessed using the GRADE approach.

Intervention	Outcome		Studies/Participants	Summary Effect Measure	Heterogeneity	GRADE
Vitamin D supplementation vs. placebo or control	All-cause mortality		7 studies/2600 participants	RR: 1.04, 95% CI: 0.83 to 1.31	I^2^ = 35%	Moderate
	Frailty levels		-			
	At least one fall		2 studies/847 participants	RR: 0.99, 95% CI: 0.82 to 1.21	I^2^ = 55%	Moderate
	Fracture		2 studies/1769 participants	RR: 0.77, 95% CI: 0.59 to 1.01	I^2^ = 0%	Low
	Muscle strength (kg)		3 studies/27 participants	MD: −0.62, 95% CI: −1.74 to 0.50	I^2^ = 0%	Very low
	Gait speed		No quantitative synthesis			
	Weight Indices		No quantitative synthesis			
	Cognitive function		No data			
	Inflammatory markers		No quantitative synthesis			
	Functional measures		No quantitative synthesis			
Multicomponent supplementation vs. placebo or control	All-cause mortality		4 studies/180 participants (only two deaths were reported in total)			Very low
	Frailty levels (mean Fried’s Frailty Phenotype score)		2 studies/86 participants	MD: −0.28, 95% CI: −0.71 to 0.16	I^2^ = 0%	Very low
	At least one fall		No quantitative synthesis			
	Fracture		-			
	Muscle strength (kg)		4 studies/153 participants	MD: 0.76, 95% CI: −1.35, 2.8	I^2^ = 31%	Very low
	Gait speed		No quantitative synthesis			
	Weight Indices	BMI	2 studies/157 participants	MD: 0.69, 95% CI: −0.78 to 2.16	I^2^ = 21%	Very low
		Body weight (kg)	4 studies/188 participants	MD: 1.23, 95% CI: −0.91 to 3.37	I^2^ = 30%	Very low
	Cognitive function (MMSE mean score)		2 studies/89 participants	MD: 1.34, 95% CI: −1.45 to 4.14	I^2^ = 0	Very low
	Inflammatory markers		No quantitative synthesis			
	Functional measures		No quantitative synthesis			

## Data Availability

Metadata are available upon reasonable request.

## Supplementary Materials

The following supporting information can be downloaded at https://www.mdpi.com/article/10.3390/healthcare13222828/s1. Supplemental Table S1: Characteristics of included studies on vitamin D supplementation; Supplemental Table S2: Characteristics of included studies on multicomponent and nicotinamide supplementation.; Supplemental Table S3. Risk of bias of included studies on vitamin D supplementation.; Supplemental Table S4. Risk of bias of included studies on multicomponent and nicotinamide supplementation.; Supplemental Figure S1: Risk of bias summary in included studies; Supplemental Figure S2: Meta-analysis of studies that reported the effect of vitamin D supplementation on fractures; Supplemental Figure S3: Meta-analysis of studies that reported the effect of vitamin D supplementation on handgrip strength in frail individuals; Supplemental Figure S4: Meta-analysis of studies that reported the effect of multicomponent supplementation on handgrip strength in frail individuals; Supplemental Figure S5: Meta-analysis of studies that reported the effect of multicomponent supplementation on BMI in frail individuals; Supplemental Figure S6: Meta-analysis of studies that reported the effect of multicomponent supplementation on body weight in frail individuals; Supplemental Figure S7: Meta-analysis of studies that reported the effect of multicomponent supplementation on Mini-Mental State Examination (MMSE) in frail individuals. PubMed/Medline Search Strategy. PRISMA Checklist [60].
