# Supplementary material for: Micronutrient Supplementation in Frailty: A Systematic Review and Meta-Analysis of Randomized Controlled Trials
Source: Healthcare (Basel). 2025 Nov 7;13(22):2828. doi: 10.3390/healthcare13222828 (PMC12652154; doi:10.3390/healthcare13222828)
Supplement: Supplementary file 1 [file healthcare-13-02828-s001.zip › healthcare-3904496-supplementary.pdf]

Supplemental Table S1: Characteristics of included studies on vitamin D supplementation.

Study	Methods	Participants	Intervention	Outcomes	Notes
**Vaes 2018 [9]**	randomized, double-blind, placebo-controlled trial	≥65 y of age, had a serum 25(OH)D concentration between 20 and 50 nmol/L, a BMI (in kg/m2) between 18.5 and 35, and who were prefrail or frail based on the frailty criteria of Fried	Arms 1 (n=26), 2 (n=26) and 3(n=26) received a capsule of 10mcg 25(OH)D3 (calfecidiol), 20 mcg Vitamin D3 (cholecalciferol) or placebo respectively. Study supplements were identical in appearance and taste	Strength test and physical performance (maximal knee-extension and knee-flexion, hand grip strength, SPPB, timed up and go test, gait speed, chair rise-5 stands, 95% ellipse), serum 25(OH)D, plasma PTH, serum calcium, urine calcium-to-creatinine ratio, muscle fiber type and size, total lean mass, safety evaluation, transcriptomic analysis. Follow-up 6 months.	
**Neelemaat 2011 [10]**	randomized controlled trial	elderly patients (inclusion criteria: ≥60 years of age, expected length of hospital admission >2 days) newly admitted to the departments of general internal medicine, rheumatology, gastroenterology, dermatology, nephrology, orthopedics, traumatology, and vascular surgery. Participants described as frail by study authors, but a frailty scale was not used.	Intervention group (n=105) received 400 IU vitamin D3 and 500 mg calcium per day for 3 months. Additional oral nutritional supplement leading to an expected increase in intake of 2520 kJ/d and 24 g protein/d and telephone counseling by a dietitian. Control group (n=105) received usual care.	Functional limitations score, Physical performance score, Physical activity score, changes in body weight and fat free mass, changes in handgrip strength.	
**Flicker 2005 [11]**	Randomized, placebo-controlled double-blind, trial	Older people resident in 60 hostels and 89 nursing homes. Participants described as frail by study authors, but a frailty scale was not used. Mean Age (83.3 ± 8.8 vs 83.6 ± 7.8). Subjects whose 25D level was less than 25 nmol/L or above 90 nmol/L were excluded.	All randomized subjects were prescribed 600 mg of elemental calcium in the form of calcium carbonate to be taken daily. Intervention group (n=313) received 10,000 IU ergocalciferol tablets once per week, which was changed to o 1000 IU ergocalciferol capsules given once daily due to discontinuation of the original product. Placebo arm received placebo identical to both supplements and	Falls, fractures, compliance with therapy.	

			in identical fashion.		
**Appel 2021 [12]**	Two-stage, Bayesian, response-adaptive randomized trial	688 participants, community dwelling, aged ≥70 years, with elevated fall risk and serum 25-hydroxyvitamin D level of 25–72.5nmol/L. 61% of the participants were pre-frail and 13 % were frail at baseline (Fried's Frailty Phenotype). 91 more randomized at the confirmatory stage.	The control group dose, 200IU/day. The intervention group was assigned to 1000, 2000 or 4000 IU/day during the dose-finding part of the study. The 1000IU/day dose was declared the best dose and participants previously randomized to 2000 and 4000IU/day were switched to 1000IU/day.	The primary outcome was time to first fall or death over two years, whichever occurred first. The secondary outcome was gait speed, although study documents mentioned that other study outcomes were secondary. Gait speed was obtained from the timed 4-meter walk component of the Short Physical Performance Battery and was measured at baseline and 3, 12, and 24 months. Rates of SAEs, serious falls (fall resulting in a fracture or dislocation or a fall associated with a hospitalization or other SAE), falls with hospitalization, and serious events potentially related to vitamin D were examined for safety. Frailty components (Fried's Frailty Phenotype) and additional information about falls (indoors, outdoors, fracture etc.) in separate publications.	
**Latham 2003 [13]**	Multicenter, randomized, controlled trial with a factorial design	Aged 65 and older, considered frail according to simple clinical measures of frailty as described by Winograd ((meets any one of the following criteria) Cerebrovascular accident Chronic and disabling illness Confusion Dependence in ADL's Depression Falls Impaired mobility Incontinence Malnutrition Polypharmacy Pressure sore Prolonged bedrest Restraints Sensory impairment Socioeconomic/family problems).	Factorial 2x2 design. Arm 1: The vitamin D intervention was given in a single oral dose. Patients received either six 1.25-mg calciferol (300,000 IU) or matching placebo tablets. Arm 2: The resistance exercise intervention consisted of a quadriceps exercise program using adjustable ankle cuff weights undertaken three times per week for	Self-rated physical health (physical component of the SF-36 questionnaire) at 3 months and falls (number of falls, number of people who fell) over the 6-month period.	Effects for each intervention are reported separately.

			10 weeks. Arm 3: Combination. Arm 4: Control		
**Gloth 1995 [14]**	Randomized, controlled intervention study	Frail homebound community-dwelling older people. Patients who were less than 65 years old or who had been outside in the previous 6 months were ineligible for this study.	All subjects received either calcium or calcium and vitamin D (400 IU per day to 100,000 IU every 3 months of ergocalciferol).	Change in Frail Elderly Functional Assessment score.	Number of participants randomized in each group not reported.
**Rizka 2018 [15]**	Double blind randomized controlled trial	Elderly subjects aged >60 years old who visited a geriatric clinic. 24.5% of the participants were pre-frail and 50.9% were frail.	Intervention group received Alphcalcidiol 0.5 mcg/d	IL-6, IL-10 and IFNγ and percentage of CD4, CD8, CD8+ CD28- T cell.	
**Bjorkman 2009 [16]**	Randomized double-blind controlled trial	Long-term inpatients aged over 65 years, chronically impaired mobility, stable general condition, and no known present disease (except osteoporosis) or medication (vitamin D supplements, glucocorticoids, antiepileptics, etc.) affecting calcium or bone metabolism.	Participants were randomized in three groups receiving cholecalciferol in Migliol oil in dose equivalent to 0 IU (n=68), 400 IU (n=77) or 1200 IU (n=73) respectively. Participants with insufficient consumption of dairy products received calcium supplementation during the intervention.	Mortality, 25(OH)Vit-d, PTH, Carboxy-terminal telopeptide of type I collagen, amino-terminal propeptide of type I procollagen, calcium, phosphorous, creatinine, GFR, albumin, CRP.	
**Meyer 2002 [18]**	Double-blinded randomized controlled trial	Frail elderly nursing home residents, mean age at baseline was 84.7 years (SD ± 7.4 years).	The intervention group (n=569) received 5 ml of ordinary cod liver oil including 2.2 µg/ml vitamin D3 daily, whereas the placebo (n=575) was ordinary cod liver oil in which vitamin D had been removed (0.1-0.2 µg/ml vitamin D3). The difference between groups was 10 µg per 5-ml dose.	Hip fracture (defined as cervical or trochanteric fracture), all nonvertebral fractures, mortality, grip strength, 25-hydroxyvitamin D (calcidiol), osteocalcin, parathyroid hormone (PTH), and ionized calcium.	
**Bischoff-Ferrari 2024 [19]**	2x2 factorial designed RCT	91 pre-frail, hypogonadal older men age 65 years and older; low serum total testosterone levels (<11.30 nmol/L); at higher risk for falling (fulfill at least 1 criteria of the Fried-based frailty criteria and/or being at least at low risk for falling based on the adapted FROP-Com screening tool.	Factorial design: intervention 1) monthly 24,000 IU vitamin D3 Intervention 2) transdermal testosterone gel	Falls, appendicular lean mass, lower extremity function and strength, and gait speed.	
**Dwimartutie 2024 [20]**	Randomized double-blinded placebo-controlled clinical trial	Oder adults (age ⩾ 60 years) with pre-frail conditions. Pre-frail was defined according to the Cardiovascular Health Study (CHS) criteria	Intervention group received cholecalciferol 4000 IU/day. All subjects were given calcium lactate 500 mg/day.	Hand grip strength walking speed, expression of VDR, IGF-1 and IL-6 in monocytes.	

Supplemental Table S2: Characteristics of included studies on multicomponent and nicotinamide supplementation.

Study	Methods	Participants	Intervention	Outcomes	Notes
**Imaoka 2016[21]**	randomized, non-blind, controlled clinical trial	Frail elderly who lived in the care facility and not received any regular supplementation of vitamin D during the previous 12 months.	The control group (n = 23) was provided usual care. The low-exercise group (n = 22) did not perform group exercise, but were provided two sessions of individualized exercise each week. The nutrition group (n = 23) was given daily oral vitamin D (900 IU), via an Isocal jelly PCF (500 IU) and a supplement (400 IU). Supplementation included natrium, kalium, magnesium, phopsphorus, iron, zinc, copper, selenium, vitamin d, niacin, vitamin-b12 . The combined group (n = 23) performed the same exercise as the low-exercise group and received the same vitamin D supplementation as the nutrition group.	Mortality, hand grip strength, 25(OH)D, skeletal muscle index, hasegawa's dementia scale, functional independence measure, falls.	Only nutrition vs control groups were included in this review (n=46).
**Bonnefoy 2003 ([22]**	factorial non blinded randomized controlled trial	Frail elderly living in retirement homes with mean age over 83 years, multiple diagnoses, several medications, and a length of stay of more than 3 years in retirement homes for the elderly.	Factorial 2x2 design. Group 1: Received nutritional supplements twice daily, consisting of proteins, carbohydrates, lipids, minerals (Ca, P, Mg, Fe) and vitamins (A, B1, B2, B5, B6, nicotinamide ,B12, C, E, D3, biotin, folic acid). Placebo for nutritional supplementation had an identical packaging. Group 2: Received moderate exercise three times weekly for 60 minutes. Weekly memory sessions served as placebo for exercise program. Group 3: Combination. Group 4: Placebo.	Changes in quadriceps muscle power, changes in fat—free mass, 6m walk time, six-stair climb time, BMI, resting metabolic rate. Assessments were done at 3 and 9 months.	Outcome measures are given for nutritional intervention (n=30) vs placebo (n=27) (effect of intervention).

**Biesek 2021 [23]**	randomized controlled clinical trial	Pre-frail older women (according to Fried's Frailty Scale) with moderate" kidney functioning (i.e., a glomerular filtration rate (GFR) of 30–60 mL/min/1.73 m^2), estimated by the Chronic Kidney Disease Epidemiology Collaboration (CKD-EPI) equation; if presented, Type II diabetes had to be compensated (<8% glycated hemoglobin); and had adequate visual acuity assessed by the Snellen card (20/70 unilateral).	Participants were divided into five groups (18 participants in each group): control (CG); exergames training (ETG); protein supplementation (PSG); exergames combined with protein supplementation (ETPSG); exergames combined with isoenergetic supplementation (ETISG). Protein supplementation group received a supplement once daily which included why protein isolate, carbohydrates, lipids, minerals (sodium, potassium, chloride, calcium, iron, phosphorus, magnesium, zinc, copper, manganese ,molybdenum , selenium, chromium, iodine) and vitamins (A, D, E, K, B1, B2, niacin, pantothenic acid, B6, folic acid, B12, biotin). Isoenergetic supplementation provided amount of kcal similar to the protein supplementation group. Exercise consisted of physical training twice a week for 12 weeks.	Frailty score after 12 weeks, bmi, lean mass, appendicular skeletal muscle mass, IL-6, 25(OH)D, handgrip strength, peak torque, food intake.	Only protein supplementation and control groups were included in this study.
**Wouters-Wesseling 2003 [28]**	Randomized, double-blind, placebo controlled trial	Frail elderly people 65 years of age or older were selected based on a body mass index (BMI) of less than 25 kg/m2 and residency in a home for the elderly or sheltered housing. A frailty scale was not used.	The intervention group (n=28) received an enriched drink contained energy (100 kcal/100 mL), protein, carbohydrate, fat and micronutrients in amounts of approximately 30% to 150% of US RDA, with higher levels of antioxidants, (sodium, potassium, chloride, calcium, phosphorus, magnesium, iron, zinc, copper, manganese, fluoride, molybdenum, selenium, chlorium, iodine, vitamins A, D, E,	Plasma antioxidant level after 6 months of supplementation. Vitamin E, Vitamin C, Trolox equivalent antioxidant capacity, uric acid, cysteine, total thiol, glutathione peroxidase.	

			K, C, B1, B2, B6, B12, carotenoids, niacin, pantothenic acid, folate). The placebo (n=27) contained no energy or micronutrients.		
**Wouters-Wesseling 2005 [29]**	Randomized, double-blind, placebo-controlled trial	Frail white persons aged 65 years or older who had a BMI less than 25 kg/m2 and resided in a home for elderly persons or sheltered housing residence.	The intervention group (n=34) received an enriched drink contained energy (100 kcal/100 mL), protein, carbohydrate, fat and micronutrients in amounts of approximately 30% to 150% of US RDA, with higher levels of antioxidants, (sodium, potassium, chloride, calcium, phosphorus, magnesium, iron, zinc, copper, manganese, fluoride, molybdenum, selenium, chlorium, iodine, vitamins A, D, E, K, C, B1, B2, B6, B12, carotenoids, niacin, pantothenic acid, folate). The placebo (n=33) contained no energy or micronutrients.	Neuropsychological tests, plasma homocysteine, plasma B12. Assessments were done after 6 months of supplementation.	
**Na 2021 [24]**	Case-controlled, double-blind, and randomized controlled trial.	Elderly at community care facilities. 73% of the participants were pre-frail and 11% were frail at baseline. Mean age 80.8±7. The frailty status was determined using the Korean version of the fatigue, resistance, ambulation, illnesses, and loss of weight scale (K-FRAIL).	Intervention group (n=31) received daily oral nutritional supplement including protein, carbohydrate, fat, minerals (sodium, calcium, phosphorus, potassium, magnesium, iron, zinc), vitamins (A, B1, B2, B6, B12, C, D, E, folic acid, niacin, pantothenic acid, biotin) for 90 days. Supplementation in control group (n=31) was similar but had lower amounts of carbohydrate, fat, protein, calcium, phosphorus, zinc, vitamins A, C, D, E.	Change in weight, BMI, arm and calf circumference, body fat, lean mass, hand grip strength, appendicular skeletal muscle mass, activities of daily living, simplified nutritional appetite questionnaire, MMSE, frailty levels, changes in dietary intake. Assessments were done after 3 months of supplementation.	
**Gosney 2008 [25]**	Double-blinded randomized controlled trial	Frail elderly residents from nursing and residential homes aged over 60 years.	The micronutrient supplement and placebo (n=37) were identical in appearance. Participants took two tablets, twice a day for 8 weeks. The	HADS anxiety score, HADS depression score, MADRS score	

		Median age was 82 years.	intervention group (n=33) received an active supplement including minerals (iron, zinc, copper, iodine, manganese, chromium, selenium, molybdenum, calcium, magnesium) and vitamins (A, D3, E, B1, B2, B6, B12, C nicotinamide, folic acid, biotin, calcium pantothenate).		
**de Jong 1999[26]**	Randomized controlled trial	Free-living frail elderly Dutch people. The following criteria were used: requirement of health care, such as home care or meals-on-wheels service; age (≥ 70 y); no regular exercise; body mass index (BMI)3 below average (≤25 kg/m2 on the basis of self-reported weight and height) or recent weight loss; no use of multivitamin supplements; and ability to understand the study procedures. A frailty scale was not used. 145 out of 217 randomized participants were analyzed.	Factorial 2x2 design. Nutrition group (n=37) received of two enriched products daily , which delivered ~100% of the Dutch recommended daily allowance (RDA) of the following vitamins: D, E, thiamin, riboflavin, B-6, folic acid, B-12 and C and ~25–100% of the Dutch RDA of the following minerals: calcium (25%), magnesium (25%), zinc (50%), iron (50%) and iodine (100%). Subjects in the control (n=34) and exercise group received the natural amount of the regular products (amount of vitamins and minerals in regular products at the highest 15% of the concentration in enriched products. Exercise group (n=35) received twice daily moderate, gradually increasing intensity. Combination group (n=39) received both interventions.	Dietary intake, blood vitamins levels, biochemical, hematological and inflammatory markers serum levels, smell, taste and appetite changes, grip strength, walking speed, chair stands, fitness score, activities of daily living, mobility score, self-care score, lean body mass, performance score, neuropsychological test, psychological wellbeing (SSWO score), subjective health, social involvement. Assessment after 17 weeks of intervention.	
**Abe 2016 [27]**	Single-blind, randomized controlled trial	Frail elderly residing in nursing home and requiring special care from a helper. A	The first group (*n* = 13) received an L-leucine (1.2 g) and cholecalciferol (20 μg)–enriched supplement with 6 g medium-chain TGs	Body weight, BMI, estimated muscle mass, hand-grip strength, walking speed, peak expiratory flow, leg	

		frailty scale was not used.	(LD + MCT). As an energy-matched control to the supplementation of MCTs, the second group (*n* = 13) received the same leucine and cholecalciferol–enriched supplement with 6 g long-chain TGs (LCTs) (LD + LCT). The control group (*n* = 12;) did not receive any supplements. The control group did not receive an energy-equivalent placebo. The L-leucine and cholecalciferol supplement also included carbohydrate, fat, sodium, thiamin, pyridoxine and cyanocobalamin.	open-close test, MMSE, Nishimura geriatric scale. Study duration was 3 months.	
**Nicotinamide**					
**Akasaka 2022 [30]**	Double-blinded, randomized controlled trial	Frail elderly (by CHS criteria) >65 years old with diabetes	Oral administration of nicotinamide mononucleotide at a dose of 250 mg/day.	Grip strength, 4-m walking time, isometric knee extensor strength, time to stand on one leg with eyes open, skeletal muscle mass index, echographic measurement of lower leg muscle, plasma glucose, HbA1c, ophthalmic examinations and the number of frailty	

Supplemental Table S3. Risk of bias of included studies on vitamin D supplementation.

Study	Random sequence Generation	Allocation concealment	Blinding of participants and personnel	Blinding of outcome assessment	Incomplete outcome data	Selective reporting	Other bias
**Vaes 2018 [9]**	Low/ Randomization in permuted blocks and stratified by sex and BMI.	Low/ Investigators blinded to allocation treatment.	Low/ Double-Blinded and study supplements were identical in appearance and taste.	Low/ Investigators blinded to treatment.	Low/ 75 out of 78 participants completed the study.	Low/ Minimal deviations from published protocol.	Low/ Falls not reported, One author has a related patent, Sponsors not reported in manuscript.
**Neelemaat 2011 [10]**	Low/ A computerized random number generator was used to assign patients, in blocks of 10.	Low/ Consecutively numbered opaque envelope containing the patients' group assignment.	High/ Participants, research assistant, and researcher were not blinded.	High/ Research assistant, and researcher were not blinded. When performing the analyses, the primary investigator was not aware of the patients' group assignment.	High/ 30 out of 105 patients in each group were lost to follow up. Even higher attrition for some outcomes.	Unclear/ Netherlands registry is no longer available.	Low/ The Netherlands Organization for Health Research and Development (ZonMw) funded the trial, project number 94506203.
**Flicker 2005 [11]**	Low/ Subjects were randomized via computer-generated lists.	Unclear/ Not described in text.	Low/Residents, institutional staff, and study staff were blinded to treatment allocation.	Low/ Institutional staff, and study staff were blinded to treatment allocation.	Low/ 10% attrition after 1 year and 15% after 2 years of study.	Unclear/ Protocol not available.	Unclear / Study supplement changed during the trial. Funding for this study was provided by (Australian) National Health and Medical Research Council (NHMRC) Project Grants 964135 and 139124 and the Victorian Health Promotion Foundation (VHPF). The NHMRC and VHPF played no role in the study design or in the collection, analysis, or interpretation of data. Dr. Stein received financial support from the Wenkart Foundation and the Royal Australasian College of Physicians Vincent Fairfax Family Foundation Research Fellowship. Supplements and

							placebos were purchased commercially, and the suppliers played no role in the study design or in the collection, analysis, or interpretation of data.
**Appel 2021 [12]**	Low/ Assignments were generated using a computer-generated random number and a web-based application.	Unclear/ Not described in text.	Low / Study personnel and participants were masked to randomized dose, occurrence of adaptations, and the transition from dose-finding to confirmatory stage.	Low/ Triple-blinded, study personnel and were masked to randomized dose, occurrence of adaptations, and the transition from dose-finding to confirmatory stage.	Low/ 779 patients randomized, data on 688 patients, 15% attrition during the 4-year duration of the trial.	Low/ All outcomes in study protocol have been reported.	Low/ The NIA encouraged several design features, including an adaptive trial to assess the efficacy and dose-response of vitamin D supplementation for fall prevention and a non-placebo control group. The NIA had no role in the collection, analysis, and interpretation of data; no role in the preparation, review, and approval of the manuscript; and no role in the decision to submit this manuscript for publication
**Latham 2003 [13]**	Low/ Computerized central randomization scheme.	Unclear/ Not described in text.	Low/ Participants and personnel blinded for Vitamin-d intervention.	Low/ "Research nurses who were blinded to the assigned treatments conducted follow-up visits at 3 and 6 months post randomization in the patients' place of residence."	Low/ 7 participants withdrawn.	Unclear/ Protocol not available.	Low/ Supported by grants from the Health Research Council of New Zealand, the Auckland University of Technology Research Fund, and a bequest from the Lenore Wilson Estate.
**Gloth 1995 [14]**	Unclear/ Not described in text.	Unclear/ Not described in text.	Unclear/ Not described in text.	Unclear/ Not described in text.	Low/ All subjects completed follow-up.	Unclear/ Protocol not available.	Low/ Funding not reported.
**Rizka 2018 [15]**	Low/ Computerized random sequence generation program.	Low/ Allocation concealment was performed.	Low/ Subjects and investigators were blinded.	Low/ Outcome assessors were blinded.	Low/ All randomized studies were analyzed.	High/ Incidence of respiratory tract infection (main	Low/ Sources of funding not reported.

						outcome in registered protocol) is not reported.	
**Bjorkman 2009 [16]**	Unclear/ Not described in text.	Unclear/ Not described in text.	Low/ Patients and ward nurses were blinded to the intervention.	Unclear/ Not described in text.	High/ 173 out of 218 participants analyzed (21% attrition).	Unclear/ Protocol not available.	Low/ The study was funded by a special governmental subsidy for health sciences research and training to Helsinki University Central Hospital.
**Meyer 2002 [18]**	High/ Before the study started, the days of the month (1-31 days) were divided randomly into group A and group B, and based on the day of birth, a participant was placed automatically in group A or group B when registered in the study database.	Unclear/ The nursing staff was not aware of the details in the allocation procedures	Low/ The participants, the nursing staff and the investigators were blinded.	Low/ The outcome assessor was not otherwise involved in the study and had no knowledge about the study participants	High/ 449 out of 1144 stopped treatment for other reasons except death.	Unclear/ Protocol not available.	Low/ Funding not reported.
**Bischoff-Ferrari 2024 [19[**	Low/ Block randomization performed centrally	Low/ Centrally performed through computer software	Low/ Participants and investigators were blinded	Low/ Outcome assessors were blinded	Low/ 8% dropout during follow-up	Low/ Outcomes reported as per protocol	High/ Early termination due to low recruitment rate
**Dwimartutie 2024 [20]**	Low/ Block randomization performed centrally	Low/ Sealed envelopes	Low/ Participants and investigators were blinded	Low/ Outcome assessors were blinded	Low/ 116/120 participants were analyzed	Low/ Outcomes reported as per protocol	

Supplemental Table S4. Risk of bias of included studies on multicomponent and nicotinamide supplementation.

Study	Random sequence Generation	Allocation concealment	Blinding of participants and personnel	Blinding of outcome assessment	Incomplete outcome data	Selective reporting	Other bias
**Imaoka 2016 [21]**	Unclear/ Not described in text.	Low/ Opaque envelopes were used	High/ Blinding was not performed.	High/ Blinding was not performed.	High/34 out of 46 participants were analyzed (26% attrition).	Unclear/ Protocol not available.	Low/ This study was funded by Nestle Health Science (Tokyo, Japan). The sponsor of the study had no role in the study design, conduct of the study, data collection, data interpretation or, preparation of the report.
**Bonnefoy 2003 [22]**	Low/ Centralized and stratified between the homes and occurred the same day before starting sessions for participants in each home, using a remote data-entry system	Unclear/ Not described in text.	High/ Blinding was not performed.	High/ Blinding was not performed.	High/42 out of 57 were analyzed (26% attrition).	Unclear/ Protocol not available.	Low/ Funding and conflicts of interest not reported.
**Biesek 2021 [23]**	Low/ Randomization into blocks was performed at randomization.com.	Low/ Described in study protocol.	High/ Personnel who carried out the interventions was blinded to the group, but participants in the control did not receive placebo.	Low/ In study protocol: “the researchers who carry out the evaluations and interventions will be blinded to the allocation of the groups and the block sizes’’.	Low/ 3 participants lost to follow up in control group and 0 in the protein supplementation group.	Low/ Outcomes reported as described in protocol.	Low
**Wouters-Wesseling 2003 [28]**	Unclear/ Not described in text.	Unclear/ Not described in text.	Unclear/ Not described in text (only double-blind reported).	Unclear/ Not described in text (only double-blind reported).	Low/ No patients were lost to follow-up.	Unclear/ Protocol not available.	Low/The study was sponsored by Numico Research B.V.
**Wouters-Wesseling 2005 [29]**	Low/Randomly assigned, in groups of four matched for body mass index.	Unclear/ Not described in text.	Unclear/ Not described in text (only double-blind reported).	Unclear/ Not described in text (only double-blind reported).	High/ 67 out of 101 patients analyzed (33% attrition).	Unclear/ Protocol not available.	Low/ Sponsored by Numico Research B.V., Wageningen, The Netherlands
**Na 2021 [24]**	Unclear/ Randomization based on sex and frailty status via stratified cluster random sampling.	Unclear/ Not described in text.	Unclear/ Only characterized as double-blind with no further information.	Unclear/ only characterized as double-blind with no further information.	Low/ 53 out of 62 subjects analyzed (14% attrition).	Unclear/ Protocol not available.	Low/ NOS-NPO® and Placebo product were provided by Deasang Corporation, Korea.

**Gosney 2008 [25]**	Low/ Random numbers generated by the Hospital Pharmacy Trials Unit.	Unclear/ Not described in text.	Low/ Participants and researchers blinded to the intervention.	Unclear/ Not described in text.	High/ 59 out of 73 participants were analyzed (20% attrition).	Unclear/ Protocol not available.	Low/ The micronutrient supplement and placebo were supplied by Recip AB Sweden. Funding and potential conflicts of interest not reported.
**de Jong 1999 [26]**	Unclear/ Randomized through selection of sealed envelopes.	Unclear/ Randomized through selection of sealed envelopes.	High/ Participants and personnel were not blinded.	High/ Outcome assessors not blinded.	High/ 145 out of 217 randomized participants were analyzed (33% attrition).	Unclear/ Protocol not available.	Low/ Supported by funds from the Dutch Dairy Foundation on Nutrition and Health, Maarssen, The Netherlands, and the Health Research Council, The Netherlands.
**Abe 2016 [27]**	Unclear/ Through shuffling.	Low/ Allocation was conducted by a person who was not a member of this study. Sealed envelopes were used.	High/ Participants were only blinded between group 1 and group 2 and personnel was not blinded.	High/ Outcome assessor for walking speed was blinded, but this is not the case for other outcomes.	Low/36 out of 38 participants analyzed.	Unclear/ Protocol not available.	Low/ TGs were provided by the Nisshin OilliO Group Ltd. (Kanagawa, Japan). Potential conflicts of interest are not reported.
**Nicotinamide**							
**Akasaka 2022 [30]**	High/ Serious imbalances in the baseline data of the two groups.	Low/ Allocation performed and kept confidential by the secretariat	Low/ Participants and investigators were blinded	Low/ Outcome assessors were blinded	Low/ 13/16 participants were analyzed	Low/ Outcomes reported as per protocol	Low/ Study funded by TEIJIN Ltd/

	Random sequence generation (selection bias)	Allocation concealment (selection bias)	Blinding of participants and personnel (performance bias)	Blinding of outcome assessment (detection bias)	Incomplete outcome data (attrition bias): All outcomes	Selective reporting (reporting bias)	Other bias
Abe 2016	?	+	-	-	+	?	+
Akasaka 2022	-	+	+	+	+	+	+
Appel 2021	+	?	+	+	+	+	+
Biesek 2021	+	+	-	+	+	+	+
Bischoff-Ferrari 2024	+	+	+	+	+	+	-
Bjorkman 2005	?	?	+	?	-	?	+
Bonnefoy 2003	+	?	-	-	-	?	+
de Jong 1999	?	?	-	-	-	?	+
Dwimartutie 2024	+	+	+	+	+	+	+
Flicker 2005	+	?	+	+	+	?	?
Gloth 1995	?	?	?	?	+	?	+
Gosney 2008	+	?	+	?	-	?	+
Imaoka 2016	?	+	-	-	-	?	+
Latham 2003	+	?	+	+	+	?	+
Meyer 2002	-	?	+	+	-	?	+
Na 2021	?	?	?	?	+	?	+
Neelemaat 2011	+	+	-	-	-	?	+
Rizka 2018	+	+	+	+	+	-	+
Vaes 2018	+	+	+	+	+	+	+
Wouters-Wesseling 2003	+	?	?	?	-	?	+
Wouters-Wesseling 2005	+	?	?	?	-	?	+

Supplemental Figure S1: Risk of bias summary in included studies

Study or Subgroup	Experimental Events	Experimental Total	Control Events	Control Total	Weight	Risk ratio M-H, Random, 95% CI	Risk of Bias A	B	C	D	E	F	G
Flicker 2005	25	313	35	312	29.7%	0.71 [0.44 , 1.16]	+	?	+	+	+	?	?
Meyer 2002	60	569	76	575	70.3%	0.80 [0.58 , 1.10]	−	?	+	+	−	?	+
**Total (95% CI)**		**882**		**887**	**100.0%**	**0.77 [0.59 , 1.01]**							
Total events:	85		111										

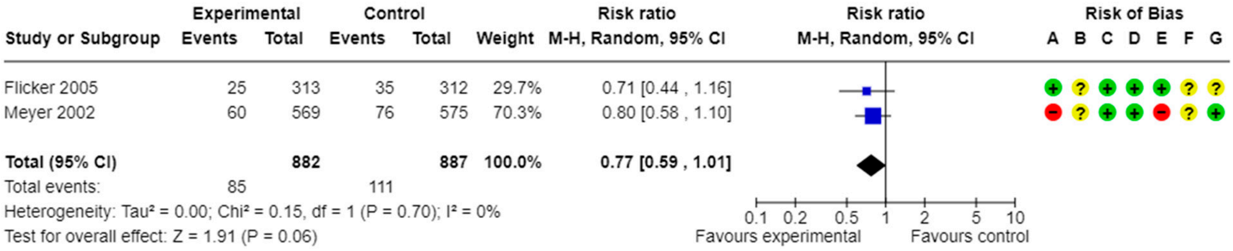

Heterogeneity: Tau² = 0.00; Chi² = 0.15, df = 1 (P = 0.70); I² = 0%
Test for overall effect: Z = 1.91 (P = 0.06)
Test for subgroup differences: Not applicable

Risk of bias legend
(A) Random sequence generation (selection bias)
(B) Allocation concealment (selection bias)
(C) Blinding of participants and personnel (performance bias)
(D) Blinding of outcome assessment (detection bias)
(E) Incomplete outcome data (attrition bias)
(F) Selective reporting (reporting bias)
(G) Other bias

Supplemental Figure S2: Meta-analysis of studies that reported the effect of vitamin D supplementation on fractures [11,18]

Study or Subgroup	Experimental Mean	Experimental SD	Experimental Total	Control Mean	Control SD	Control Total	Weight	Mean difference IV, Random, 95% CI	Risk of Bias A	B	C	D	E	F	G
Dwimartutie 2024	0.76	2.53	58	0.24	2.1	58	52.4%	0.52 [-0.33 , 1.37]	+	+	+	+	+	+	+
Meyer 2002	0.4	3.8	23	1.6	4	28	13.5%	-1.20 [-3.35 , 0.95]	−	?	+	+	−	?	+
Neelemaat 2011	0.2	5.6	65	1	6.7	53	12.3%	-0.80 [-3.06 , 1.46]	+	+	−	−	−	?	+
Vaes 2018	1.1	3.36	52	1.3	3.46	26	21.8%	-0.20 [-1.81 , 1.41]	+	+	+	+	+	+	+
**Total (Wald[a])**			**198**			**165**	**100.0%**	**-0.03 [-0.87 , 0.81]**							

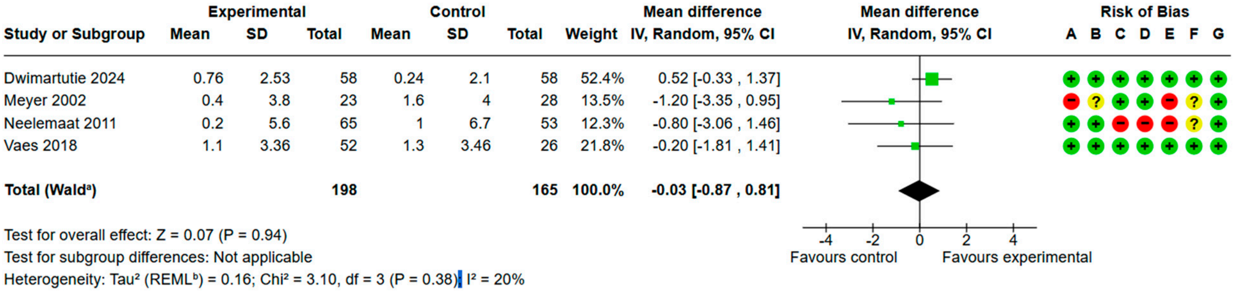

Test for overall effect: Z = 0.07 (P = 0.94)
Test for subgroup differences: Not applicable
Heterogeneity: Tau² (REML[b]) = 0.16; Chi² = 3.10, df = 3 (P = 0.38); I² = 20%

Footnotes
[a]CI calculated by Wald-type method.
[b]Tau² calculated by Restricted Maximum-Likelihood method.

Risk of bias legend
(A) Random sequence generation (selection bias)
(B) Allocation concealment (selection bias)
(C) Blinding of participants and personnel (performance bias)
(D) Blinding of outcome assessment (detection bias)
(E) Incomplete outcome data (attrition bias)
(F) Selective reporting (reporting bias)
(G) Other bias

Supplemental Figure S3: Meta-analysis of studies that reported the effect of vitamin D supplementation on handgrip strength in frail individuals[9,10,18,20]

Study or Subgroup	Experimental Mean	Experimental SD	Experimental Total	Control Mean	Control SD	Control Total	Weight	Mean difference IV, Random, 95% CI	A	B	C	D	E	F	G
Abe 2016	11.17	3.97	24	9.8	5.5	10	22.7%	1.37 [-2.39 , 5.13]	?	+	-	-	+	?	+
Biesek 2021	21.4	4.5	17	20.1	5.4	15	25.5%	1.30 [-2.17 , 4.77]	+	+	-	+	+	+	+
Imaoka 2016	10.2	5.3	17	13.5	7.6	17	17.9%	-3.30 [-7.70 , 1.10]	?	+	-	-	-	?	+
Na 2021	13.1	5.5	28	11	4.8	25	33.9%	2.10 [-0.67 , 4.87]	?	?	?	?	+	?	+
**Total (95% CI)**			**86**			**67**	**100.0%**	**0.76 [-1.35 , 2.87]**							

Heterogeneity: Tau² = 1.43; Chi² = 4.32, df = 3 (P = 0.23); I² = 31%
Test for overall effect: Z = 0.71 (P = 0.48)
Test for subgroup differences: Not applicable

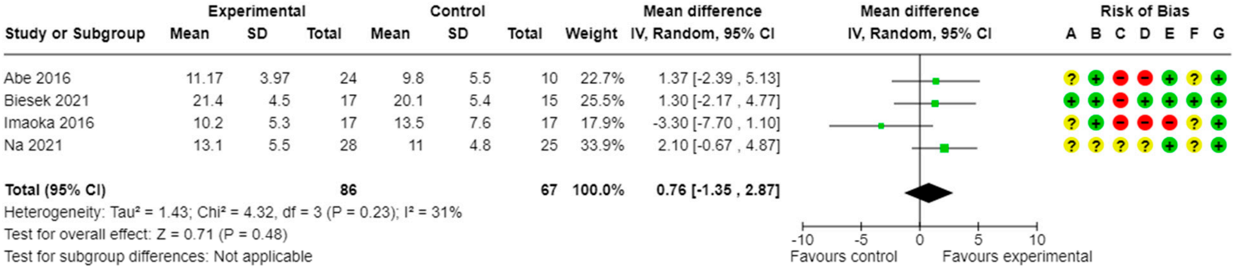

Risk of bias legend
(A) Random sequence generation (selection bias)
(B) Allocation concealment (selection bias)
(C) Blinding of participants and personnel (performance bias)
(D) Blinding of outcome assessment (detection bias)
(E) Incomplete outcome data (attrition bias)
(F) Selective reporting (reporting bias)
(G) Other bias

Supplemental Figure S4: Meta-analysis of studies that reported the effect of multicomponent supplementation on handgrip strength in frail individuals[21,23,24,27]

Study or Subgroup	Experimental Mean	Experimental SD	Experimental Total	Control Mean	Control SD	Control Total	Weight	Mean difference IV, Random, 95% CI	A	B	C	D	E	F	G
Abe 2016	19.2	2.672	25	19.3	2.7	11	47.2%	-0.10 [-2.01 , 1.81]	?	+	-	-	+	?	+
Na 2021	24.1	3.4	28	22.7	3.2	25	52.8%	1.40 [-0.38 , 3.18]	?	?	?	?	+	?	+
**Total (95% CI)**			**53**			**36**	**100.0%**	**0.69 [-0.78 , 2.16]**							

Heterogeneity: Tau² = 0.24; Chi² = 1.27, df = 1 (P = 0.26); I² = 21%
Test for overall effect: Z = 0.92 (P = 0.36)
Test for subgroup differences: Not applicable

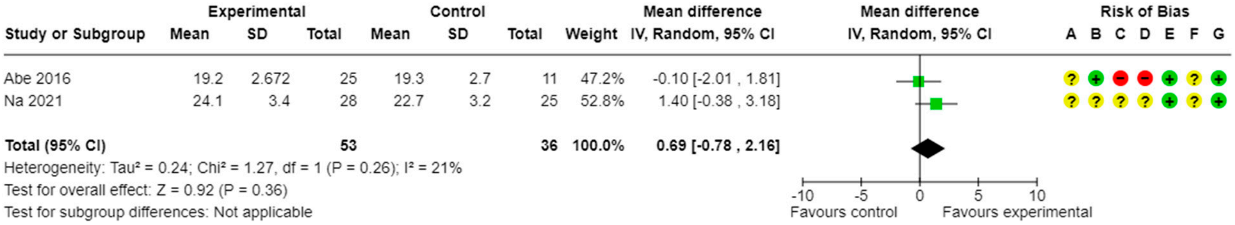

Risk of bias legend
(A) Random sequence generation (selection bias)
(B) Allocation concealment (selection bias)
(C) Blinding of participants and personnel (performance bias)
(D) Blinding of outcome assessment (detection bias)
(E) Incomplete outcome data (attrition bias)
(F) Selective reporting (reporting bias)
(G) Other bias

Supplemental Figure S5: Meta-analysis of studies that reported the effect of multicomponent supplementation on BMI in frail individuals [24,27]

	Experimental			Control				Mean difference	Risk of Bias						
Study or Subgroup	Mean	SD	Total	Mean	SD	Total	Weight	IV, Random, 95% CI	A	B	C	D	E	F	G
Abe 2016	43.18	7.42	25	42.9	6.1	11	16.3%	0.28 [-4.35 , 4.91]	?	+	−	−	+	?	+
Biesek 2021	67	11.3	16	66.5	12.2	15	6.1%	0.50 [-7.79 , 8.79]	+	+	−	+	+	+	+
de Jong 1999	0.2	1.2	35	-0.3	1.7	33	63.4%	0.50 [-0.20 , 1.20]	?	?	−	−	−	?	
Na 2021	57.7	10.1	28	51.8	8.7	25	14.2%	5.90 [0.84 , 10.96]	?	?	?	?	+	?	+
**Total (95% CI)**			**104**			**84**	**100.0%**	**1.23 [-0.91 , 3.37]**							

Heterogeneity: Tau² = 1.76; Chi² = 4.31, df = 3 (P = 0.23); I² = 30%
Test for overall effect: Z = 1.13 (P = 0.26)
Test for subgroup differences: Not applicable

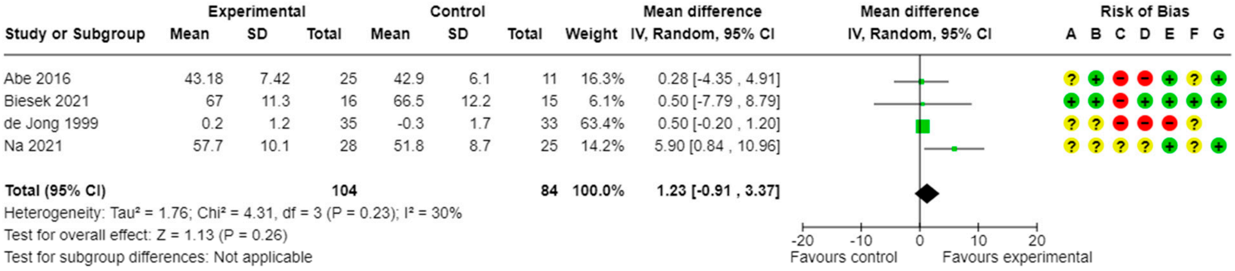

Risk of bias legend
(A) Random sequence generation (selection bias)
(B) Allocation concealment (selection bias)
(C) Blinding of participants and personnel (performance bias)
(D) Blinding of outcome assessment (detection bias)
(E) Incomplete outcome data (attrition bias)
(F) Selective reporting (reporting bias)
(G) Other bias

Supplemental Figure S6: Meta-analysis of studies that reported the effect of multicomponent supplementation on body weight in frail individuals[23,24,26,27]

	Experimental			Control				Mean difference	Risk of Bias						
Study or Subgroup	Mean	SD	Total	Mean	SD	Total	Weight	IV, Random, 95% CI	A	B	C	D	E	F	G
Abe 2016	18.4	8.44	25	14.9	10	11	17.1%	3.50 [-3.27 , 10.27]	?	+	−	−	+	?	+
Na 2021	15.7	6.2	28	14.8	5.2	25	82.9%	0.90 [-2.17 , 3.97]	?	?	?	?	+	?	+
**Total (95% CI)**			**53**			**36**	**100.0%**	**1.34 [-1.45 , 4.14]**							

Heterogeneity: Tau² = 0.00; Chi² = 0.47, df = 1 (P = 0.49); I² = 0%
Test for overall effect: Z = 0.94 (P = 0.35)
Test for subgroup differences: Not applicable

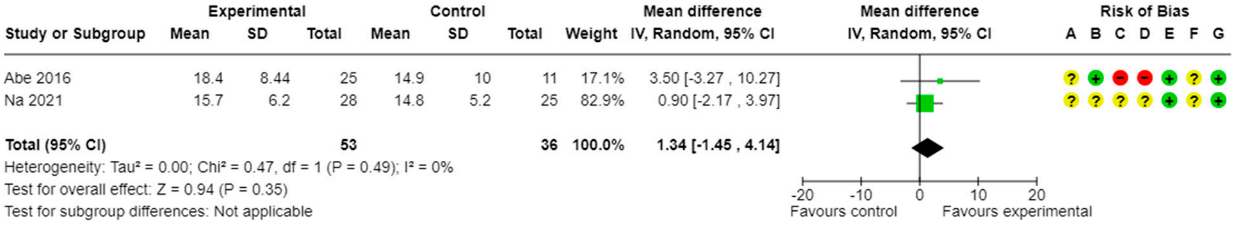

Risk of bias legend
(A) Random sequence generation (selection bias)
(B) Allocation concealment (selection bias)
(C) Blinding of participants and personnel (performance bias)
(D) Blinding of outcome assessment (detection bias)
(E) Incomplete outcome data (attrition bias)
(F) Selective reporting (reporting bias)
(G) Other bias

Supplemental Figure S7: Meta-analysis of studies that reported the effect of multicomponent supplementation on Mini-Mental State Examination (MMSE) in frail individuals [24,27]

PubMed/Medline Search Strategy

#1 "Frail Elderly"[Mesh]

#2 "Frailty"[Mesh]

#3 frail*[tiab]

#4 pre-frail*[tiab]

#5 prefrail*[tiab]

#6 "frailty syndrome"[tiab]

#7 "physical frailty"[tiab]

#8 #1 OR #2 OR #3 OR #4 OR #5 OR #6 OR #7

#9 "Dietary Supplements"[Mesh]

#10 "Micronutrients"[Mesh]

#11 "Vitamins"[Mesh]

#12 "Minerals"[Mesh]

#13 "Vitamin D"[Mesh]

#14 vitamin*[tiab]

#15 mineral*[tiab]

#16 micronutrient*[tiab]

#17 multivitamin*[tiab]

#18 "nutritional supplement*"[tiab]

#19 "dietary supplement*"[tiab]

#20 lycopene[tiab]

#21 ascorbic[tiab]

#22 tocopherol[tiab]

#23 retinol[tiab]

#24 folate[tiab]

#25 carotenoid*[tiab]

#26 beta-carotene[tiab]

#27 selenium[tiab]

#28 pyridoxine[tiab]

#29 iron[tiab]

#30 nicotinamide[tiab]

#31 #9 OR #10 OR #11 OR #12 OR #13 OR #14 OR #15 OR #16 OR #17 OR #18 OR #19 OR #20 OR #21 OR #22 OR #23 OR #24 OR #25 OR #26 OR #27 OR #28 OR #29 OR #30

#32 "Randomized Controlled Trial"[Publication Type]

#33 randomized[tiab]

#34 randomised[tiab]

#35 randomly[tiab]

#36 placebo[tiab]

#37 trial[tiab]

#38 #32 OR #33 OR #34 OR #35 OR #36 OR #37

#39 aged[Mesh]

#40 elderly[tiab]

#41 "older adult*"[tiab]

#42 "older people"[tiab]

#43 "older population"[tiab]

#44 #39 OR #40 OR #41 OR #42 OR #43

#45 #8 AND #31 AND #38 AND #44

Section and Topic	Item #	Checklist item	Location where item is reported
**TITLE**			
Title	1	Identify the report as a systematic review.	Page 1 Title
**ABSTRACT**			
Abstract	2	See the PRISMA 2020 for Abstracts checklist.	Page 1 Abstract
**INTRODUCTION**			
Rationale	3	Describe the rationale for the review in the context of existing knowledge.	Page 2 Introduction
Objectives	4	Provide an explicit statement of the objective(s) or question(s) the review addresses.	Page 2 Introduction
**METHODS**			
Eligibility criteria	5	Specify the inclusion and exclusion criteria for the review and how studies were grouped for the syntheses.	Section 2.1
Information sources	6	Specify all databases, registers, websites, organisations, reference lists and other sources searched or consulted to identify studies. Specify the date when each source was last searched or consulted.	Section 2.2
Search strategy	7	Present the full search strategies for all databases, registers and websites, including any filters and limits used.	Supplementary Files
Selection process	8	Specify the methods used to decide whether a study met the inclusion criteria of the review, including how many reviewers screened each record and each report retrieved, whether they worked independently, and if applicable, details of automation tools used in the process.	Section 2.3
Data collection process	9	Specify the methods used to collect data from reports, including how many reviewers collected data from each report, whether they worked independently, any processes for obtaining or confirming data from study investigators, and if applicable, details of automation tools used in the process.	Section 2.3
Data items	10a	List and define all outcomes for which data were sought. Specify whether all results that were compatible with each outcome domain in each study were sought (e.g. for all measures, time points, analyses), and if not, the methods used to decide which results to collect.	Section 2.3
	10b	List and define all other variables for which data were sought (e.g. participant and intervention characteristics, funding sources). Describe any assumptions made about any missing or unclear information.	Section 2.3
Study risk of bias assessment	11	Specify the methods used to assess risk of bias in the included studies, including details of the tool(s) used, how many reviewers assessed each study and whether they worked independently, and if applicable, details of automation tools used in the process.	Section 2.4
Effect measures	12	Specify for each outcome the effect measure(s) (e.g. risk ratio, mean difference) used in the synthesis or presentation of results.	Section 2.5
Synthesis methods	13a	Describe the processes used to decide which studies were eligible for each synthesis (e.g. tabulating the study intervention characteristics and comparing against the planned groups for each synthesis (item #5)).	Section 2.5
	13b	Describe any methods required to prepare the data for presentation or synthesis, such as handling of missing summary statistics, or data conversions.	Section 2.5
	13c	Describe any methods used to tabulate or visually display results of individual studies and syntheses.	Section 2.5
	13d	Describe any methods used to synthesize results and provide a rationale for the choice(s). If meta-analysis was performed, describe the model(s), method(s) to identify the presence and extent of statistical heterogeneity, and software package(s) used.	Section 2.5
	13e	Describe any methods used to explore possible causes of heterogeneity among study results (e.g. subgroup analysis, meta-regression).	Not performed/paucity of data
	13f	Describe any sensitivity analyses conducted to assess robustness of the synthesized results.	Not performed/

Section and Topic	Item #	Checklist item	Location where item is reported
			paucity of data
Reporting bias assessment	14	Describe any methods used to assess risk of bias due to missing results in a synthesis (arising from reporting biases).	Not performed/ small number of studies
Certainty assessment	15	Describe any methods used to assess certainty (or confidence) in the body of evidence for an outcome.	Section 2.4
**RESULTS**			
Study selection	16a	Describe the results of the search and selection process, from the number of records identified in the search to the number of studies included in the review, ideally using a flow diagram.	Section 3.1
	16b	Cite studies that might appear to meet the inclusion criteria, but which were excluded, and explain why they were excluded.	None relevant
Study characteristics	17	Cite each included study and present its characteristics.	Section 3.2
Risk of bias in studies	18	Present assessments of risk of bias for each included study.	Section 3.3 and supplementary files
Results of individual studies	19	For all outcomes, present, for each study: (a) summary statistics for each group (where appropriate) and (b) an effect estimate and its precision (e.g. confidence/credible interval), ideally using structured tables or plots.	Section 3.4
Results of syntheses	20a	For each synthesis, briefly summarise the characteristics and risk of bias among contributing studies.	Section 3.4
	20b	Present results of all statistical syntheses conducted. If meta-analysis was done, present for each the summary estimate and its precision (e.g. confidence/credible interval) and measures of statistical heterogeneity. If comparing groups, describe the direction of the effect.	Section 3.4
	20c	Present results of all investigations of possible causes of heterogeneity among study results.	Not performed/paucity of data
	20d	Present results of all sensitivity analyses conducted to assess the robustness of the synthesized results.	Not performed/ paucity of data
Reporting biases	21	Present assessments of risk of bias due to missing results (arising from reporting biases) for each synthesis assessed.	Not performed/ small number of studies
Certainty of evidence	22	Present assessments of certainty (or confidence) in the body of evidence for each outcome assessed.	Section 3.4, Table 1
**DISCUSSION**			
Discussion	23a	Provide a general interpretation of the results in the context of other evidence.	Section 4, page 14

Section and Topic	Item #	Checklist item	Location where item is reported
	23b	Discuss any limitations of the evidence included in the review.	Section 4 page 15
	23c	Discuss any limitations of the review processes used.	Section 4 page 15
	23d	Discuss implications of the results for practice, policy, and future research.	Section 4 page 15
**OTHER INFORMATION**			
Registration and protocol	24a	Provide registration information for the review, including register name and registration number, or state that the review was not registered.	Section 2.1.1
	24b	Indicate where the review protocol can be accessed, or state that a protocol was not prepared.	Section 2.1.1
	24c	Describe and explain any amendments to information provided at registration or in the protocol.	Sectoin 2.1.1
Support	25	Describe sources of financial or non-financial support for the review, and the role of the funders or sponsors in the review.	Page 16
Competing interests	26	Declare any competing interests of review authors.	Page 16
Availability of data, code and other materials	27	Report which of the following are publicly available and where they can be found: template data collection forms; data extracted from included studies; data used for all analyses; analytic code; any other materials used in the review.	Page 16

Prisma Checklist with location of items reported [60]
